# Supplementary material for: Epiperipatus puri sp. nov., a new velvet worm from Atlantic Forest in Southeastern Brazil (Onychophora, Peripatidae)
Source: PeerJ. 2023 Oct 2;11:e15384. doi: 10.7717/peerj.15384 (PMC10552768; doi:10.7717/peerj.15384)
Supplement: Supplemental Information 1 [file peerj-11-15384-s001.docx]

Table. Data from vouchers of *Epiperipatus puri* sp. nov. and closely related species (see Figure 4). Information from Costa et al. (2020). Sequences deposited in GenBank.

| Species/morphotype | Collection Number | Voucher | Locality | COI | 12S | 16S | 18S |
| --- | --- | --- | --- | --- | --- | --- | --- |
| *Epiperipatus machadoi* | MNRJ0043 | ony-008 | Brazil, Minas Gerais, Caratinga | MN905632 | MN639360 | MN544108 | - |
| *Epiperipatus ohausi* | MNRJ0056 | ony-011 | Brazil, Rio de Janeiro, Nova Iguaçu | MN905634 | MN639361 | MN544109 | MN705441 |
| *Epiperipatus ohausi* | MNRJ0058 | ony-010 | Brazil, Rio de Janeiro, Nova Iguaçu | MN905633 | - | - | - |
| *Epiperipatus puri* | MNRJ0087 | ony-037 | Brazil, Rio de Janeiro, Cachoeiras de Macacu | MN905649 | - | MN544125 | - |
| *Epiperipatus puri* | MNRJ0088 | ony-036 | Brazil, Rio de Janeiro, Cachoeiras de Macacu | MN905648 | MN639375 | MN544124 | MN705445 |
| *Epiperipatus puri* | MNRJ0093 | ony-065 | Brazil, Rio de Janeiro, Cachoeiras de Macacu | MN905672 | MN639394 | MN544148 | - |
| *Epiperipatus* sp | MNRJ0016 | ony-032 | Brazil, Espírito Santo, Cariacica | - | - | MN544122 | - |
| *Epiperipatus* sp | MNRJ0100 | ony-153 | Brazil, Espírito Santo, Santa Leopoldina | - | MN639449 | MN544194 | MN705490 |
| *Epiperipatus* sp5 | MNRJ0059 | ony-012 | Brazil, Rio de Janeiro, Guapimirim | MN905635 | - | MN544110 | - |
| *Epiperipatus* sp11 | MNRJ0042 | ony-027a | Brazil, Espírito Santo, Santa Teresa | MN905643 | MN639368 | MN544117 |  |
| *Epiperipatus* sp11 | MNRJ0042 | ony-027b | Brazil, Espírito Santo, Santa Teresa | MN905644 | MN639369 | MN544118 |  |
| *Epiperipatus* sp11 | MNRJ0042 | ony-027c | Brazil, Espírito Santo, Santa Teresa | MN905645 | MN639370 | MN544119 |  |
| *Epiperipatus* sp11 | MNRJ0046 | ony-003 | Brazil, Espírito Santo, Santa Teresa | MN905628 | - | MN544104 | - |
| *Epiperipatus* sp13 | MNRJ0018 | ony-030a | Brazil, Espírito Santo, Linhares | - | MN639372 | MN544121 | - |
| *Epiperipatus* sp13 | MNRJ0018 | ony-030b | Brazil, Espírito Santo, Linhares | - | MN639373 | - | - |
| *Epiperipatus* sp13 | MNRJ0020 | ony-029 | Brazil, Espírito Santo, Linhares | - | MN639371 | MN544120 | - |
| *Epiperipatus* sp13 | MNRJ0023 | ony-048 | Brazil, Espírito Santo, Pinheiros | MN905657 | MN639383 | MN544133 | - |
| *Epiperipatus* sp13 | MNRJ0026 | ony-049 | Brazil, Espírito Santo, Pinheiros | HG531958 | MN933779 | MN933788 | - |
| *Epiperipatus* sp14 | MNRJ0016 | ony-032 | Brazil, Espírito Santo, Cariacica | - | - | MN544122 | - |
